# Supplementary material for: Cognitive reserve is associated with education, social determinants, and cognitive outcomes among older American Indians in the Strong Heart Study
Source: Commun Psychol. 2025 Jan 28;3:14. doi: 10.1038/s44271-025-00198-6 (PMC11775147; doi:10.1038/s44271-025-00198-6)
Supplement: Supplementary file 2 — Reporting Summary [file 44271_2025_198_MOESM2_ESM.pdf]

nature portfolio

Corresponding author(s): DBPR COMMSPSYCHOL-24-0035

Last updated by author(s): 2024-July 8

Reporting Summary

Nature Portfolio wishes to improve the reproducibility of the work that we publish. This form provides structure for consistency and transparency in reporting. For further information on Nature Portfolio policies, see our Editorial Policies and the Editorial Policy Checklist.

Statistics

For all statistical analyses, confirm that the following items are present in the figure legend, table legend, main text, or Methods section.

n/a	Confirmed	
☐	☒	The exact sample size (*n*) for each experimental group/condition, given as a discrete number and unit of measurement
☐	☒	A statement on whether measurements were taken from distinct samples or whether the same sample was measured repeatedly
☐	☒	The statistical test(s) used AND whether they are one- or two-sided<br>*Only common tests should be described solely by name; describe more complex techniques in the Methods section.*
☐	☒	A description of all covariates tested
☐	☒	A description of any assumptions or corrections, such as tests of normality and adjustment for multiple comparisons
☐	☒	A full description of the statistical parameters including central tendency (e.g. means) or other basic estimates (e.g. regression coefficient) AND variation (e.g. standard deviation) or associated estimates of uncertainty (e.g. confidence intervals)
☐	☒	For null hypothesis testing, the test statistic (e.g. *F*, *t*, *r*) with confidence intervals, effect sizes, degrees of freedom and *P* value noted<br>*Give P values as exact values whenever suitable.*
☒	☐	For Bayesian analysis, information on the choice of priors and Markov chain Monte Carlo settings
☒	☐	For hierarchical and complex designs, identification of the appropriate level for tests and full reporting of outcomes
☐	☒	Estimates of effect sizes (e.g. Cohen's *d*, Pearson's *r*), indicating how they were calculated

Our web collection on statistics for biologists contains articles on many of the points above.

Software and code

Policy information about availability of computer code

Data collection	No code used to collect data.
Data analysis	Analysis code used available on request as described in the manuscript.

For manuscripts utilizing custom algorithms or software that are central to the research but not yet described in published literature, software must be made available to editors and reviewers. We strongly encourage code deposition in a community repository (e.g. GitHub). See the Nature Portfolio guidelines for submitting code & software for further information.

Data

Policy information about availability of data

All manuscripts must include a data availability statement. This statement should provide the following information, where applicable:

- Accession codes, unique identifiers, or web links for publicly available datasets
- A description of any restrictions on data availability
- For clinical datasets or third party data, please ensure that the statement adheres to our policy

Data from the Strong Heart Study and its ancillary studies cannot be made publicly available, but can be accessed per study and Tribal policies, as described here: https://strongheartstudy.org.

Human research participants

Policy information about studies involving human research participants and Sex and Gender in Research.

Reporting on sex and gender	This research is population based. Sexes are represented as M/F according to self-reported identity.
Population characteristics	This research is population based. Participant characteristics are represented in proportions according to self-report or measurement, as collected from the cohort population. Please see Table 1 for accounting of all characteristics used in these analyses.
Recruitment	This research is population based. The baseline study was recruited from communities in 1989, representing 67% of all adults in the age range of interest (45-95) at that time. The current study was recruited using contact rosters from that original exam.
Ethics oversight	There are 11 IRB, RRB, and tribal council involved in this research. They are named in previous reports, and cited in this report. Full ethics oversight was conducted of all study procedures, all participants provided written informed consent, all tribes approved the study dissemination report.

Note that full information on the approval of the study protocol must also be provided in the manuscript.

Field-specific reporting

Please select the one below that is the best fit for your research. If you are not sure, read the appropriate sections before making your selection.

☒ Life sciences ☐ Behavioural & social sciences ☐ Ecological, evolutionary & environmental sciences

For a reference copy of the document with all sections, see nature.com/documents/nr-reporting-summary-flat.pdf

Life sciences study design

All studies must disclose on these points even when the disclosure is negative.

Sample size	This is a population based study of individuals living in 11 tribes and communities aged 65 and older, and includes all available willing participants.
Data exclusions	No data were excluded or ommitted.
Replication	There are no experimental findings. The quality control of the observational study data is described in great detail in previous reports, and cited. The quality control for the data specifically for this analysis is also included in this report.
Randomization	Not applicable.
Blinding	Not applicable.

Reporting for specific materials, systems and methods

We require information from authors about some types of materials, experimental systems and methods used in many studies. Here, indicate whether each material, system or method listed is relevant to your study. If you are not sure if a list item applies to your research, read the appropriate section before selecting a response.

Materials & experimental systems

n/a	Involved in the study	
☒	☐	Antibodies
☒	☐	Eukaryotic cell lines
☒	☐	Palaeontology and archaeology
☒	☐	Animals and other organisms
☒	☐	Clinical data
☒	☐	Dual use research of concern

Methods

n/a	Involved in the study	
☒	☐	ChIP-seq
☒	☐	Flow cytometry
☐	☒	MRI-based neuroimaging

Magnetic resonance imaging

Experimental design

Design type	observational
Design specifications	cohort study
Behavioral performance measures	No behavioral component

Acquisition

Imaging type(s)	structural MRI
Field strength	1.5 Tesla
Sequence & imaging parameters	1) sagittal T1-weighted localizer, 2) co-registered 5mm axial-T1, 3) 5 mm axial-T2, and 4) 5mm axial-T2* susceptibility-weighted images in the anterior commissure/posterior commissure plane, 5) 3mm axial fluid-attenuated inversion recovery (FLAIR) images, and 6) 1.5mm sagittal T1-weighted volumetric gradient echo images. Additional details regarding repetition time, echo time, inversion time, slice thickness, slice spacing, acquisition matrix, number excitations, echo train, flip angle, and field of view provided in prior reports, as cited
Area of acquisition	Whole brain

Diffusion MRI ☐ Used ☒ Not used

Preprocessing

Preprocessing software	FreeSurfer V5.3
Normalization	skull stripping using cortical reconstruction implemented in version 5.3 of the FreeSurfer image analysis suite includes motion correction, removal of non-brain tissue using a hybrid watershed-surface deformation procedure, automated Talairach transformation, segmentation of the subcortical white matter and deep gray matter volumetric structures, intensity normalization, tessellation of the gray matter-white matter boundary, automated topology correction, and surface deformation following intensity gradients to place optimally the gray matter-white matter and gray matter-CSF borders at the location where the greatest shift in intensity defines the transition to the other tissue class. Therefore, brain volume measurement includes cerebellum but not ventricles, CSF, and dura.
Normalization template	First, a training set of manually segmented images included with the software were parameterized as surface meshes and then modeled as a point distribution, by searching through linear combination of shape modes of variation for the most probable shape instance, given the observed intensities in a T1-weighted image. To accommodate partial-volume scans, the range of registration parameters was limited to −5 to +20 degrees. Images were checked at key intermediate points, such as after registrations and skull stripping, and adjustments were made at those points.
Noise and artifact removal	Images were visually checked at intermediate points in the process for gross misregistrations or processing failures. Such problems were corrected manually. Skull stripping using cortical reconstruction by voxel based parcellation of T1 images
Volume censoring	Only images which exceeded FOV were censored; these usually did not pass QC

Statistical modeling & inference

Model type and settings	The volume of WMH was calculated by segmenting 3mm FLAIR images (sequence 5) using the Fuzzy Lesion Extractor (FLEX) technique. FLEX uses a dynamic threshold, identifying candidate WMH voxels that are brighter than the gray matter, followed by false positive detection. After noise reduction filtering and removal of hyperintense voxels, a two-class unsupervised fuzzy C-means clustering algorithm is applied to separate remaining voxels into either brain tissue or background-CSF. Using this method, WMH voxels are outliers in both of these clusters. After masking out these hyperintense voxels based on the membership grade from fuzzy C-means clustering, the final segmentation threshold was determined on a slice-by-slice basis. This was done by creating histograms of the voxel intensities in the masked and unmasked FLAIR images, and setting the threshold at the intensity of the first bin that contained unequal numbers of voxels. To detect false positives, the brain was masked with the thresholded white matter template (white matter probability = .41), and hyperintensities that were not connected in 3D to the thresholded template were removed.
Effect(s) tested	Continuous volumes estimates as mm3 or ML, standardized to intracranial space (as % IC volume)

Specify type of analysis: ☒ Whole brain ☐ ROI-based ☐ Both

Statistic type for inference (See Eklund et al. 2016)	regional volume as % IC volume used continuously in statistical analysis
Correction	as % IC volume

Models & analysis

n/a	Involved in the study
☒	☐ Functional and/or effective connectivity
☒	☐ Graph analysis
☐	☒ Multivariate modeling or predictive analysis

Multivariate modeling and predictive analysis	Summary (N %, mean SD, med IQR); graphical (scatter, box, histogram, heat map); correlation/association (Pearson, ANOVA); mediation (causal analysis). All fully described in methods.
